# Book Notices

**Published:** 1865-11

**Authors:** 


### Book Notices.

THE PRACTICE OF MEDICINE AND SURGERY APPLIED TO THE DISEASES AND ACCIDENTS INCIDENT TO WOMEN. By WM. H. BYFORD, A.M., M.D., author of "A Treatise on the Chronic Inflammations and Displacements of the Unimpregnated Uterus," and Professor of Obstetrics and Diseases of Women and Children, in the Chicago Medical College. Philadelphia: LINDSAY & BLAKISTON. 1865.

This is an elegantly executed volume, of 556 pages, done up in the best style of the publishers. The type, paper, and binding are all excellent.

The work embraces thirty chapters, devoted to the consideration of the following topics, namely:—Diseases and Accidents of the Labia and Perineum; Diseases of the Vulva; Vaginitis; Menstruation and its Disorders; Menorrhagia; Dysmenorrhœa; Misplaced Menstruation; Acute Inflammation of the Unimpregnated Uterus; Chronic Inflammation of the Uterus and Cervex; Complications of Inflammation of the Cervex; General and Local Treatment; Treatment of Sub-mucous Inflammation; Perimetritis; Displacements, their Philosophy and Treatment; Diseased Deviations of Involution of the Uterus; Cancer of the Uterus; Tumors of the Uterus; Ovarian Tumors; Diseases of the Mammæ; Phlegmasia Alba Dolens, or Crural Phlebitis; Puerperal Fever; Stomatitis Materni, or Nursing Sore Mouth. The author is an experienced writer, an able teacher in his department, and has embodied in the present work the results of a wide field of practical observation. We have not had time to read its pages critically, but freely commend it to all our readers, as one of the most valuable practical works issued from the Americon press.

For sale by S. C. Griggs & Co., Chicago. Price $5.

---

The Student's Book of Cutaneous Medicine and Diseases of the Skin. By Erasmus Wilson, F.R.S. New York: William Wood & Co., 61 Walker Street. 1865.

This is a good-sized volume, of 445 pages; good type, fair paper and binding. It is, in fact, an abridgment or condensation of Wilson's well-known voluminous work on cutaneous diseases. The first chapter is devoted to a consideration of the anatomy and physiology of the skin; and the second to its pathology, and the classification of its diseases. The remaining twenty-two chapters are devoted to the detailed description and treatment of the different varieties of cutaneous disease.

Though we do not like the author's manner of grouping or classifying the diseases of the skin, in all respects, yet the work is a plain practical treatise, admirably adapted to the wants of medical students. It is sufficiently full, yet not too voluminous, for a good text-book. We fully endorse the closing paragraph of the author's preface. He says:—"Our aim has been to simplify, to endeavor to restore to general medicine, a department of much interest and importance, and, by furnishing the student with a clear view of these diseases, to remove them from the narrow sphere of Specialism to the wider and nobler field of Catholic Medicine."

For sale by W. B. Keen & Co., Lake Street, Chicago.

---

Lectures on the Diseases of the Stomach, with an Introduction on its Anatomy and Physiology. By Williams Brinton, M.D., F.R.S., Physician to St. Thomas' Hospital. From the second English edition. Philadelphia: Lea & Blanchard. 1865.

This is a full-sized octavo volume of 302 pages; embracing eight lectures, besides the preliminary chapter on the Anatomy and Physiology of the Stomach. The first chapter relates to the Symptoms of Gastric Diseases generally; the second, to the Morbid Anatomy, or *Post Mortem* Appearances; the third, to

Ulcer of the Stomach; the fourth, to Cancer; the fifth, to Cirrhotic Inflammation, or Plastic Linitis of the Stomach; the sixth, to Dyspepsia; the seventh, to Gastric Phthisis; and the eighth, to Gout in the Stomach. The work is a valuable addition to our medical literature.

For sale by W. B. KEEN & Co., Lake Street, Chicago.

---

The following works have been received, and will be more fully noticed hereafter.

THE USE OF THE LARYNGOSCOPE IN DISEASES OF THE THROAT, &c. By MORRILL MACKENZIE, M.D., London, M.R.C.P. Philadelphia: LINDSAY & BLAKISTON. 1865.

RESEARCHES ON THE MEDICAL PROPERTIES AND APPLICATIONS OF PROTOXIDE OF NITROGEN, OR LAUGHING GAS. By GEO. J. ZIEGLER, M.D., &c. Philadelphia: LIPPINCOTT & Co. 1865.

LECTURES ON INFLAMMATION, BEFORE THE COLLEGE OF PHYSICIANS OF PHILADELPHIA. By JOHN H. PACKARD, M.D., &c. Philadelphia: J. B. LIPPINCOTT & Co. 1865.

LECTURES ON FEVER, &c. By A. P. MERRILL, M.D., &c. New York: HARPER & BROTHERS, Publishers. 1865.

PHYSICIAN'S VISITING LIST, DIARY, AND BOOK OF ENGAGEMENTS, FOR 1866. Philadelphia: LINDSAY & BLAKISTON. 1865.

For sale by W. B. KEEN & Co., Lake Street, Chicago.

